# The Effects of In Utero Fetal Hypoxia and Creatine Treatment on Mitochondrial Function in the Late Gestation Fetal Sheep Brain

**DOI:** 10.1155/2022/3255296

**Published:** 2022-01-29

**Authors:** Anna Maria Muccini, Nhi T. Tran, Nadia Hale, Matthew McKenzie, Rod J. Snow, David W. Walker, Stacey J. Ellery

**Affiliations:** ^1^The Ritchie Centre, Hudson Institute of Medical Research, Clayton, Victoria, Australia; ^2^Department of Obstetrics & Gynaecology, Monash University, Clayton, Victoria, Australia; ^3^Faculty of Health Science, RMIT University, Bundoora, Victoria, Australia; ^4^School of Life and Environmental Sciences, Faculty of Science, Engineering, And Built Environment, Deakin University, Geelong, Victoria, Australia; ^5^Centre for Innate Immunity and Infectious Diseases, Hudson Institute of Medical Research, Clayton, Victoria, Australia; ^6^Department of Molecular and Translational Science, Monash University, Clayton, Victoria, Australia; ^7^Institute for Physical Activity & Nutrition, School of Exercise and Nutrition Sciences, Deakin University, Geelong, Victoria, Australia

## Abstract

Near-term acute hypoxia in utero can result in significant fetal brain injury, with some brain regions more vulnerable than others. As mitochondrial dysfunction is an underlying feature of the injury cascade following hypoxia, this study is aimed at characterizing mitochondrial function at a region-specific level in the near-term fetal brain after a period of acute hypoxia. We hypothesized that regional differences in mitochondrial function would be evident, and that prophylactic creatine treatment would mitigate mitochondrial dysfunction following hypoxia; thereby reducing fetal brain injury. Pregnant Border-Leicester/Merino ewes with singleton fetuses were surgically instrumented at 118 days of gestation (dGa; term is ~145 dGA). A continuous infusion of either creatine (*n* = 15; 6 mg/kg/h) or isovolumetric saline (*n* = 16; 1.5 ml/kg/h) was administered to the fetuses from 121 dGa. After 10 days of infusion, a subset of fetuses (8 saline-, 7 creatine-treated) were subjected to 10 minutes of umbilical cord occlusion (UCO) to induce a mild global fetal hypoxia. At 72 hours after UCO, the fetal brain was collected for high-resolution mitochondrial respirometry and molecular and histological analyses. The results show that the transient UCO-induced acute hypoxia impaired mitochondrial function in the hippocampus and the periventricular white matter and increased the incidence of cell death in the hippocampus. Creatine treatment did not rectify the changes in mitochondrial respiration associated with hypoxia, but there was a negative relationship between cell death and creatine content following treatment. Irrespective of UCO, creatine increased the proportion of cytochrome *c* bound to the inner mitochondrial membrane, upregulated the mRNA expression of the antiapoptotic gene *Bcl2,* and of *PCG1-α,* a driver of mitogenesis, in the hippocampus. We conclude that creatine treatment prior to brief, acute hypoxia does not fundamentally modify mitochondrial respiratory function, but may improve mitochondrial structural integrity and potentially increase mitogenesis and activity of antiapoptotic pathways.

## 1. Introduction

Acute in utero fetal hypoxia is a common complication of pregnancy [1], often leading to long-term neurological impediments for surviving infants, and sometimes even to perinatal death [2, 3]. As such, understanding the pathophysiology triggered by hypoxia remains at the forefront of clinical interest. This is particularly important for developing appropriate and timely treatment options [4, 5]. One consideration in the development of new therapies is that brain injury arising from in utero hypoxia is not uniform with some regions displaying greater vulnerability to hypoxia [6], including the hippocampus [7], cortical grey matter, and white matter [8, 9].

A high constitutive metabolic demand has been identified as a characteristic that increases the susceptibility to hypoxic injury in specific regions of the fetal brain [10]. The high metabolic requirement of the developing brain uses aerobic respiration along with other pathways to sustain normal growth and development [11]. It is therefore reasonable to suggest that optimal mitochondrial function is important for brain development throughout gestation and at the transition to neonatal life [12, 13]. Emerging evidence has shown that mitochondria are central to the hypoxic-ischemic injury response, and mitochondrial compromise is thought to underpin the secondary and tertiary stages of energy failure that culminates in perinatal brain injury, thereby making mitochondria a target for therapeutic intervention [14–17]. Indeed, studies have shown that hypoxic-ischemic injury impairs mitochondrial respiration in the adult rat brain [18, 19], alters mitogenesis in the neonatal rat brain [20, 21], and decreases mitochondrial respiration following a mild hypoxic insult in neonatal mice [22]. However, only a few studies have focused on mitochondrial function in the brain prior to birth [23]. One study conducted on cortical neurons isolated from embryonic day 18 rats found ischemia-like conditions induced breakdown of mitochondrial structure and disruption of fusion processes, but did not assess the capacity of mitochondria to support ATP production after hypoxic exposure [23]. As such, there is a gap in our knowledge of how fetal brain mitochondria respond in the context of in utero fetal hypoxia at a functional level.

Mitochondrial function is a highly regulated process controlled by an array of signaling mechanisms [24]. Therefore, to determine the state of mitochondrial function in response to hypoxia several key aspects need to be taken into consideration. These include the role of mitochondrial respiration and oxidative phosphorylation (OXPHOS); as well as changes in mitochondrial and electron transport chain (ETC) complex densities, processes of fission and fusion, and overall mitochondrial structure.

There are several intracellular pathways that can support ATP production under hypoxic conditions. Phosphorylated creatine is one substrate that is used to buffer ATP levels even under basal conditions [25]. In the brain, creatine and phosphocreatine concentrations differ across regions roughly in accordance to the basal (constitutive) energy turnover characteristic of each region [26]. There is growing evidence that increased creatine availability prior to periods of oxygen starvation can support energy metabolism and protect the brain [27–29]. Indeed, creatine supplementation has been shown to be an effective prophylactic antenatal treatment against hypoxia in a spiny mouse model of birth asphyxia [29, 30]. This neuroprotective effect of creatine administered prior to hypoxic stress is thought to arise because mitochondrial function and structure is preserved for longer during the period of oxygen deprivation [31]. However, this has not been tested in large animal models nor at a region-specific level within the fetal brain.

We have recently characterized a near-term fetal sheep model of in utero hypoxia where the fetus was treated with a direct continuous infusion of creatine prior to, during, and following global hypoxia produced by a 10-minute umbilical cord occlusion (UCO) [32]. These experiments were conducted at ~130 dGA, when fetal the sheep is considered to be at a level of physiological maturity equivalent to the near-term human fetus [33]. The primary objective of the UCO was to produce a brief period of mild hypoxia, a condition that can arise transiently in late gestation. The creatine treatment timeline was chosen to determine if creatine could serve as an effective prophylactic treatment in the event of unpredictable, and potentially unidentifiable hypoxic insult to the fetus—a not uncommon occurrence in clinical settings. This study found that fetal creatine treatment increased plasma concentrations 5-fold after 4 days of continuous infusion, with no effect on basal blood gases, pH, glucose or lactate concentrations, nor on the fetal response during UCO, or during the early recovery stage (up to 24 hours after the insult). Creatine treated fetuses did however display changes to arterial oxygenation in 24-72 hours after UCO [32]. Importantly, at 13 days after commencing creatine treatment, significant creatine loading was detected in the grey matter, hippocampus, thalamus, and striatum [32].

The aim of the current study was to test the efficacy of prior creatine loading on mitochondrial respiratory activity and the incidence of neuropathology in key regions of the fetal brain, using tissue samples collected from [32]. We hypothesized that regional differences in mitochondrial function would be evident, and that prophylactic creatine treatment would mitigate mitochondrial dysfunction following hypoxia, thereby reducing fetal brain injury, an outcome that has been documented in this fetal model on several previous occasions [7, 34–36]. Tissue samples were collected at 72 hours after this hypoxic event, on the reasoning that this would allow sufficient time for the primary, secondary, and tertiary waves of energy failure, described in detail elsewhere [37–40], to evolve as a result not only of the hypoxia itself but also reperfusion that is known to cause of fetal and perinatal brain injury.

## 2. Materials and Methods

### 2.1. Animals and Surgery

Full details on the surgical preparation and methodology for this fetal sheep experiment have been previously published [32]. The animals included in that study provide the tissue samples studied in detail in this paper. In brief, 28 Border-Leicester/Merino time-mated pregnant ewes with singleton fetuses were included in this study. At 118 dGA, each ewe was placed under general anesthesia using 2% isoflurane in oxygen, and under strict sterile conditions, a mid-line abdominal incision was made to expose the uterus. The fetus was then partially exteriorized via a uterotomy to expose the fetal head and forelimbs. A medially directed polyvinyl catheter was inserted into the left brachial artery for obtaining arterial blood samples and another into the left brachial vein for administering creatine or saline solutions by infusion. The fetus was then withdrawn further until the umbilical cord could be seen, and an inflatable silastic vascular occluder (type-OC 16 mm, In Vivo Metric, Healdsburg, CA, USA) was placed around it and tethered to the fetal abdomen. The fetal uterine and maternal abdominal incisions were then repaired, with all the catheters exteriorized from the maternal abdomen through a small incision (1-2 cm) in the right flank of the ewe. Anesthesia was then withdrawn, and the ewe allowed to recover before placing her in a stall in the company of other sheep.

Fetuses were then randomly allocated to one of four experimental groups: saline-control, creatine-control, saline-UCO, or creatine-UCO, where control refers to non-UCO. All creatine-treated fetuses were subjected to a constant, intravenous infusion of creatine (6 mg.kg^−1^.h^−1^ diluted in saline and delivered at 1.5 mL/h) for a total of 13 days (from 121 to 134 dGA), while the control groups were infused with isovolumetric saline (1.5 mL.h^−1^; 0.9% NaCl, pH 7.4). Preliminary studies by Walker et al. confirmed this dosing regimen significantly increased tissue creatine content in multiple fetal organs, including the brain (supplementary Table S1). At 131 dGA, 10 days after starting the creatine or saline infusion, the subset of fetuses randomly allocated to the UCO groups (i.e., saline-UCO or creatine-UCO) underwent a 10-minute period of UCO. At 72 hours after the UCO, the ewe and fetus were humanely killed using an overdose of pentobarbitone injected intravenously to the ewe, as previously described [32]. Final analyses included tissues collected from saline-control (*n* = 4 male, *n* = 4 female), creatine-control (*n* = 4 male, n = 3 female), saline-UCO (*n* = 5 male, *n* = 3 female), and creatine-UCO (*n* = 1 male, *n* = 7 female) fetuses.

### 2.2. Tissue Collection

The fetal cerebral hemispheres were extracted from the skull and hemisected along the midline immediately after the ewe and fetus had been euthanized. Samples of the following brain regions of the left hemisphere were dissected and snap frozen in liquid nitrogen for molecular analyses: cortical grey matter, subcortical white matter, and the hippocampus. Fresh tissue samples were also collected from the cortical grey matter, periventricular white matter, and the hippocampus for immediate ex vivo assessment of mitochondrial respiratory function. The right cerebral hemisphere was left intact and immersion fixed in 4% paraformaldehyde (PFA; Merck in 0.1 mol/L phosphate buffer) for at least 24 hours, after which time it was transversely cut into 5 mm-thick sections, moved to macroembedding cassettes, and then fixed for a further 5 days in 4% PFA before being processed to paraffin.

### 2.3. High Resolution Respirometry

Respirometry was carried out on fetal brain regions according to methodology described elsewhere [41–43]. Briefly, a sample of tissue from each brain region of interest was finely minced with a scalpel blade and placed in freshly prepared ice-cold homogenization buffer (5 mM HEPES pH 7.6, 210 mM mannitol, 70 mM sucrose, 1 mM EDTA, 2 mg/mL BSA, 0.5 mM PMSF). Mitochondrial isolation was then achieved by differential centrifugation and resuspension of the pellet in fresh isolation buffer (5 mM HEPES pH 7.6, 210 mM mannitol, 70 mM sucrose, 1 mM EDTA). Mitochondrial protein concentration (mg/mL) was calculated using the formula (*A*_280nm_ − *A*_310nm_)/1.05 × 600 [44]. Oxygen consumption was measured using the Oroboros Oxygraph-2 k (Oroboros Instruments, Austria) in respiratory medium (225 mM Mannitol, 75 mM sucrose, 10 mM KCl, 10 mM Tris-HCl-pH 7.2, 5 mM KH_2_PO_4_ pH 7.2). For each region of interest, an aliquot of isolated mitochondrial suspension (adjusted to 400 *μ*g/mL) was added to the 2 mL chamber to measure state I respiration (no substrate, no ADP). Substrates were then introduced sequentially into the chamber as follows: 6.5 mM succinate (Sigma-Aldrich #S2378) to measure state IV (high substrate, no ADP) and then 0.25 mM ADP (Sigma-Aldrich #A5285) to measure state III (high substrate and high ADP) [42, 43]. Oxygen concentration was calculated using a two-point calibration of 100% O_2_ (room air, R1) and 0% O_2_ (depleted with 2 g/mL sodium hydrosulfite, R0). Oxygen consumption was calculated as oxygen flux per second per mg of isolated mitochondrial suspension (pmol.s^−1^.mg^−1^) and reported as the average of three replicate runs for each region and for each animal.

### 2.4. Western Blotting

Frozen tissue samples (~10 mg) were homogenized in RIPA buffer (Thermo-Scientific # 89901) containing a protease inhibitor (EDTA-free protease inhibitor cocktail, Sigma-Aldrich) for 2 hours at 4°C on a shaker. Samples were centrifuged, and protein concentration was determined using Pierce BCA Protein Assay Kit (#23225, Thermo Scientific, Australia) following the manufacturer's instructions. Aliquots of samples at a final protein concentration of 10 *μ*g for hippocampal samples and 40 *μ*g for white matter samples were separated by SDS-PAGE using BioRad stain-free precast protein gel (4–15% Mini-PROTEAN® TGX Stain-Free™ Protein Gels, #4568084, Life Science Research, BioRad, Australia). Before proceeding to transfer, the gel was activated using a stain-free enabled ChemiDoc (ChemiDoc™ Imaging System, #12003153, BioRad, Australia) for 5 minutes according to manufacturer instructions.

After transfer, the total protein was visualized using a ChemiDoc [45]. Proteins were then transferred onto a PVDF membrane using a BioRad Mini Trans-Blot cell (BioRad, Australia), blocked for 30 minutes in 2.5% skim milk powder in TBS, and incubated overnight in 1 : 5000 OXPHOS antibody cocktail (OxPhos Rodent WB Antibody Cocktail, #45-8099, Invitrogen, Australia). Finally, membranes were incubated for 1 hour with secondary antibody (Pierce, goat anti-mouse IgG, ThermoFisher, #31430) before being scanned using the ChemiDoc Imaging System. Image Lab software (version 6, BioRad) was used to determine the optical density (OD) of each band of interest. To account for any interblot variability, ODs for each band were normalized to the OD of a positive control (healthy rat heart protein) run on each gel. ODs for each band were then further normalized to total protein for each sample [45]. Data are expressed relative to the saline-control group.

### 2.5. Histological Analyses

Immunofluorescence and immunohistochemistry were performed on paraffin sections from parietal/temporal lobes (Cx6 and Cx7), which included cortical grey matter, subcortical white matter, and two hippocampus regions: CA1-CA3 and the dentate gyrus [34].

The colocalization of COX-IV (located in the mitochondrial inner membrane) and cytochrome *c* oxidase was used to assess mitochondrial integrity via immunofluorescence [46]. Antigen retrieval was performed by heating the slides in citrate buffer (pH 6.0) for 7 minutes. Following antigen retrieval, sections were incubated in warmed 0.3 M glycine (30 minutes at room temperature on a shaker) to block nonspecific binding. Sections were then incubated at 4°C overnight with anti-COX-IV (1 : 200, Abcam #33985, US) and anti-cytochrome *c* oxidase antibody (1 : 500, Abcam #13575, US) primary antibodies. Sections were subsequently incubated with the appropriate secondary antibodies, goat anti-rabbit IgG (Red fluorophore; 1 : 200, Vector Laboratories, Burlingame, CA, #BA-1000) and anti-mouse IgG (Green fluorophore; 1 : 500, anti-mouse, #A11001) for 3 hours at 4°C. Slides were imaged using an Olympus FV1200 Confocal Microscope at ×100 magnification. For each region of interest, 12 nonoverlapping images (field size 126.98 *μ*m^2^ per image) were captured, except for the dentate gyrus where 6 images were captured (due to the smaller size of this region). The ratio of cytochrome *c* oxidase colocalized with COX-IV/nonmitochondrial cytochrome *c* oxidase was analyzed using ImageJ software (Fuji). In the same set of images, the percentage of mitochondrial COX-IV fluorescence intensity per tissue area was determined as a proxy measurement of mitochondrial density, using a protocol optimized according to previously described methodology [47].

Immunohistochemistry was used to identify cell death (apoptosis). Eight *μ*m thick paraffin sections were reacted with ApopTag® Peroxidase In Situ Apoptosis Detection, which labels terminal deoxynucleotidyl transferase dUTP (TUNEL), according to manufacturer's instructions (Millipore Corporation, Australia, Code no. S7100). Sections were pretreated with 20 *μ*g/mL Proteinase K (15 min at 37°C). ApopTag stained sections were digitally scanned (Image Scope, Aperio Technologies Inc., Germany) and analyzed at ×40 magnification. An outline of the total area of each region of interest was manually traced, and positively stained ApopTag cells were manually counted by an observer blinded to the experimental group. Data are reported as cells per area in each region (cells/mm^2^).

### 2.6. Total RNA Extraction and cDNA Preparation

Total RNA was extracted from 60 to 80 mg of frozen tissue samples using the PureLink RNA Mini Kit, according to manufacturer instructions (Life Technologies #121830181). A spectrophotometer (Nanodrop, Thermoscientific, USA) was used to measure RNA purity and concentration. Complementary DNA (cDNA) was prepared from 1 to 5 *μ*g of sample using the SuperScript III transcriptase and primers according to manufacturer's instructions (Promega, Madison, Wisconsin). Cycling conditions were as follows: stage 1, 60 minutes at 42°C; stage 2, 5 minutes at 99°C; and stage 3, 5 minutes at 4°C.

### 2.7. Gene Analysis

The mRNA expression of genes associated with cellular and mitochondrial markers of cerebral injury (*CASP3*, *BAK*, *BAX*, *BCL2*, *ANGTP2*, *HIF1α*, *BAX:BCL2*), mitochondria structure and function (*TRMT11:B2M*, *PGC1α*, *TFAM*, *DNML1*, *FIS1*, *MFF*, *OPA1*, *MFN1*, *MFN2*, *MIEF1*), energy-sensing pathways (*SIRT1*, *SIRT3*, *ERRα*, *PRKAA1*, *PRKAA2*), and creatine metabolism (*SLC6A8, CKMT*) was quantitated using the Fluidigm Dynamic Array (Biomark, Gene expression, #BMK-M-96.96). Gene expression was determined and validated using TaqMan® FAM™ labeled probes (list gene name and catalogue # in supplementary Table S2). Note: there are no commercially available TaqMan® FAM™ labeled probes for electron complex chain 1 (CI) in sheep. As such, this marker was omitted from analysis. Cycle threshold (Ct) values were generated for each gene of interest and were analyzed using the *^ΔΔ^*CT method. The expression of all genes was expressed relative to the “geomean” of the average of three housekeeping genes *RPS16*, *RPL32*, and *OAZ1* as previously described [48]. Results are reported as fold change relative to the saline-control group.

### 2.8. Statistical Analysis

All statistical analyses were conducted using GraphPad Prism. Data was tested for normality. Statistical significance was set at *p* ≤ 0.05. Differences between treatment groups were determined using a two-way ANOVA to assess the effects of UCO (*P*_UCO_) and creatine treatment (*P*_TREAT_) and to then determine if there was an interaction between UCO and treatment (*P*_INT_). Where a significant interaction was observed, posthoc analysis was performed using Tukey's multiple comparison test. A mixed-effects analysis was also undertaken to ascertain whether mitochondrial respiration differed by fetal brain region, irrespective of UCO. Correlation analysis was performed using Spearman's rank correlation coefficient. Data are presented as mean ± SEM.

## 3. Results

For this cohort of fetal sheep, UCO for 10 mins resulted in arterial hypoxia, hypercapnia, acidemia, and lactic acidosis, as detailed elsewhere [32]. There were no differences between the saline and creatine treated fetuses in the systemic response to UCO; however, 13 days of creatine treatment did lead to significant creatine loading in the grey matter, hippocampus, thalamus, and striatum [32].

### 3.1. Mitochondrial Respiration

In the fetuses that received only saline infusion and did not undergo UCO, basal mitochondrial respiration differed by brain region, with state I respiration in the hippocampus being 30% higher compared to that recorded in the periventricular white matter (*p* = 0.02) and 36% higher compared to the cortical grey matter (*p* = 0.05). Changes to mitochondrial respiration in response to UCO also differed by fetal brain region.

**Figure 1:**
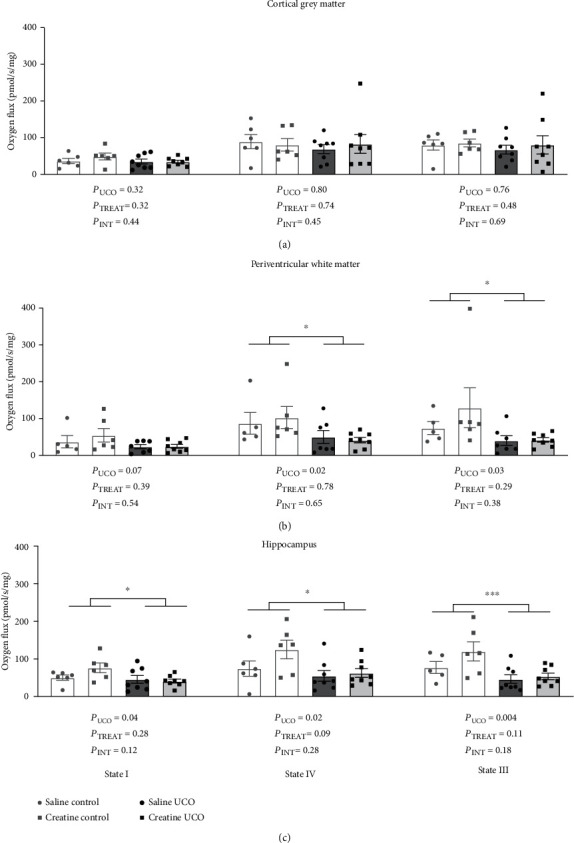
Mitochondrial respiration reported for state I, state IV, and state III respiration in cortical grey matter (a), periventricular white matter (b), and hippocampus (c). Statistics presented are two-way ANOVA and Tukey multiple comparisons. Data is shown as mean ± SEM. Saline-control (*n* = 5 − 8), creatine-control (*n* = 6), saline-UCO (*n* = 7 − 8), and creatine-UCO (*n* = 7 − 8). Significance denoted by ^∗^ refers to *p* ≤ 0.05, and ^∗∗∗^ refers to *p* ≤ 0.01.

In the cortical grey matter, neither UCO nor creatine treatment altered any of the mitochondrial respiration parameters when measured at 72 hours after the UCO (Figure 1(a)). In contrast, state IV and state III respiration in the periventricular white matter were significantly decreased with UCO (Figure 1(b); *P*_UCO_ = 0.02 and *P*_UCO_ = 0.03, respectively). Creatine treatment did not affect these outcomes.

In the hippocampus (Figure 1(c)), a decrease across all parameters of mitochondrial respiration was observed at 72 hours post-UCO. State I respiration decreased by 11% in the saline-UCO and by 19% in the creatine-UCO cohorts compared to saline-controls (*P*_UCO_ = 0.04). State IV respiration was reduced by 30% in the saline-UCO and by 11% in the creatine-UCO fetuses compared to the saline-control fetuses (*P*_UCO_ = 0.02). The UCO also decreased state III respiration by 29% in the saline-UCO and by 12% in the creatine-UCO fetuses, compared to saline-controls (*P*_UCO_ = 0.004). There was no direct effect of creatine treatment on state I or state IV respiration in the hippocampus nor were any interactions between creatine treatment and UCO observed.

### 3.2. OXPHOS mRNA and Protein Quantification

To determine if the changes in mitochondrial respiration observed in the hippocampus and white matter were associated with alterations in key ETC complexes, mRNA and protein expression were also measured (representative images of western blots are provided in Supplementary Figures S1 & S2).

**Figure 2:**
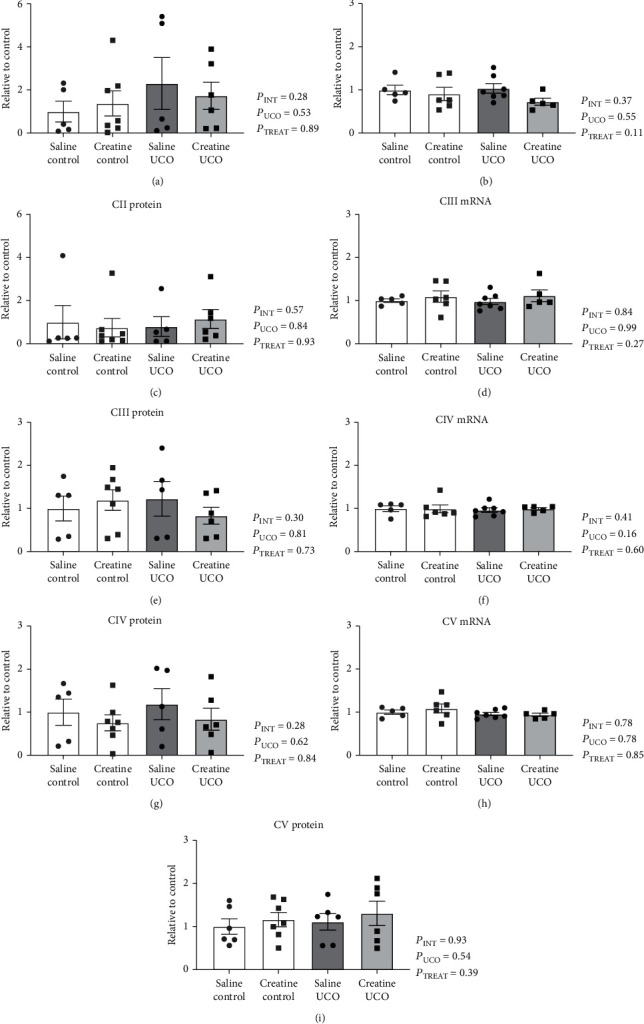
ETC CI-CV mRNA and protein expression in the hippocampus. Expression normalized to geomean of housekeeping genes or total protein stain, respectively, and expressed relative to saline-control fetuses. Two-way ANOVA and Tukey multiple comparisons. Data are mean ± SEM. Saline-control (*n* = 4 − 6), creatine-control (*n* = 6 − 7), saline-UCO (*n* = 5 − 7), and creatine-UCO (*n* = 5 − 6). Significance denoted by ^∗^ refers to *p* ≤ 0.05.

**Figure 3:**
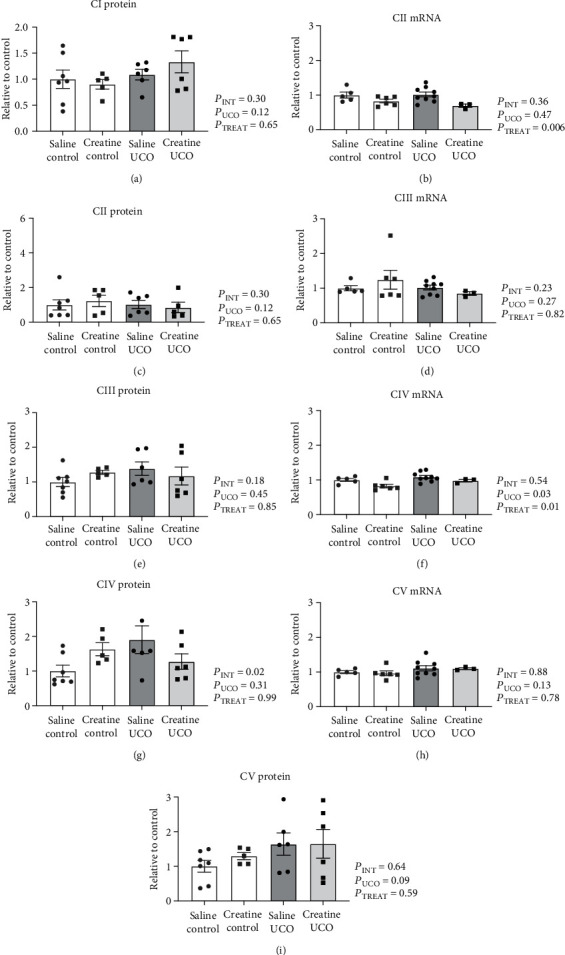
ETC CI-CV mRNA and protein expression in subcortical white matter. Expression normalized to geomean of housekeeping genes or total protein stain, respectively, and expressed relative to saline-control fetuses. Two-way ANOVA and Tukey multiple comparisons. Data are mean ± SEM. Saline-control (*n* = 5 − 6), creatine-control (*n* = 5 − 6), saline-UCO (*n* = 5 − 8), and creatine-UCO (*n* = 3 − 6). Significance denoted by ^∗^ refers to *p* ≤ 0.05.

In the hippocampus, UCO and creatine treatment did not alter the mRNA expression of ETC complexes *CII*, *CIII*, *CIV* or *CV*, or protein expression of CI, CII, CIII, CIV, or CV (Figure 2). In the subcortical white matter, creatine treatment significantly downregulated *CII* mRNA expression in both the creatine-control and creatine-UCO fetuses (Figure 3(b); *P*_TREAT_ = 0.006); however, this was not confirmed at a protein level. *CIV* mRNA expression was upregulated by UCO (Figure 3(f); *P*_UCO=_ 0.03) and decreased with creatine treatment (Figure 3(f); *P*_TREAT=_ 0.01). A significant interaction effect (*P*_INT=_ 0.02) was identified for the CIV protein expression; however, multiple comparisons analysis did not reveal any specific differences between treatment groups (Figure 3(g)). *CIII* and *CV* mRNA and protein expression were not significantly altered by either UCO or creatine treatment (Figure 3).

### 3.3. Mitochondrial Density and Cytochrome c Release

**Figure 4:**
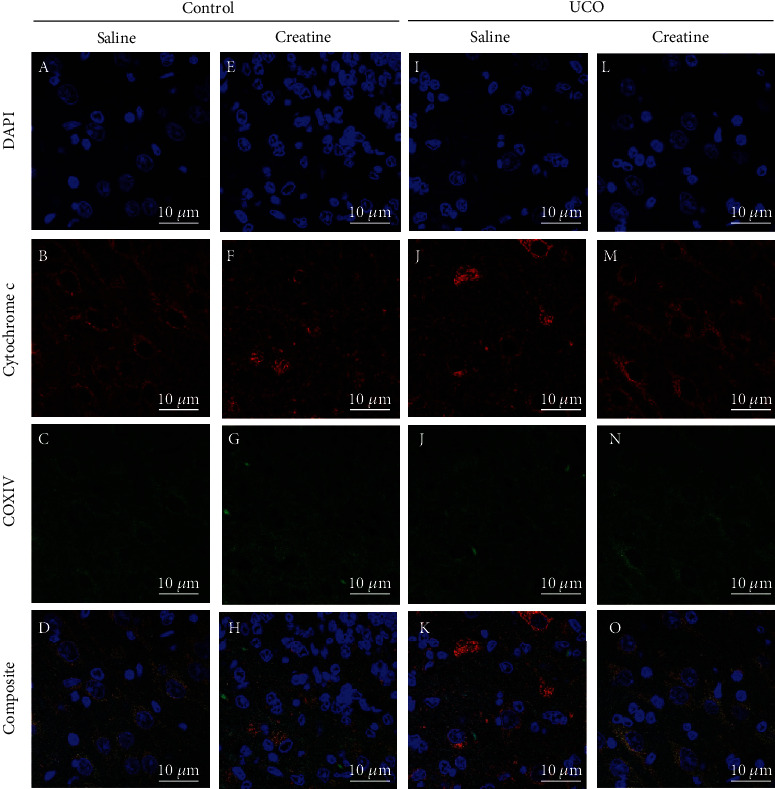
Representative images of the CA1-CA3 region of the hippocampus. Image obtained using Olympus FV1200 Confocal Microscope at ×100 magnification, oil objective. Fluorescence intensity of DAPI nuclei staining (blue), cytochrome *c* oxidase (red), COXIV (green), and composite image of all three channels in the CA1-CA3 region of the hippocampus. Colocalization of COXIV and cytochrome *c* (yellow punctate staining in composite image) indicates mitochondrial integrity. Images acquired from the saline-control (a)–(d), creatine-control (e)–(h), saline-UCO (i)–(l), and creatine-UCO (m)–(o) fetuses.

**Figure 5:**
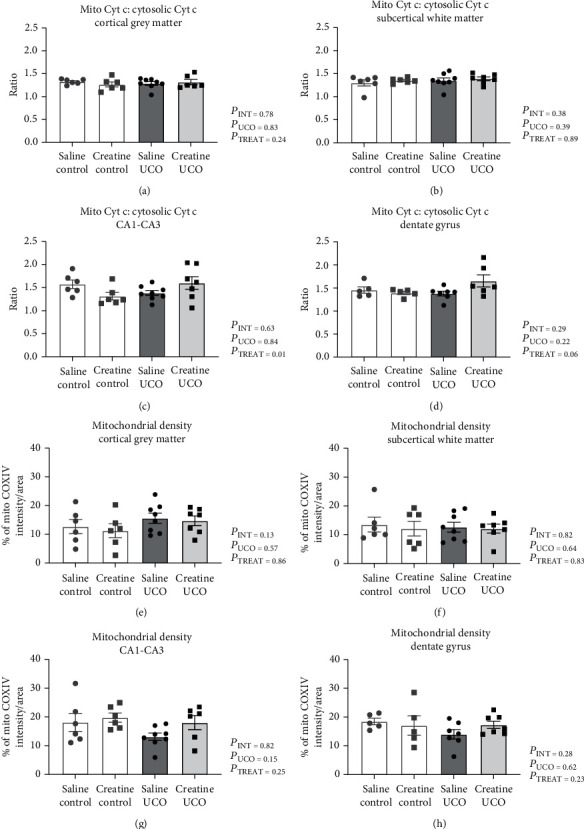
Mitochondrial density and cytochrome *c* release. The ratio of mitochondrial cytochrome *c* to nonmitochondrial cytochrome *c* in the cortical grey matter (a), subcortical white matter (b), hippocampus CA1-CA3 (c), and dentate gyrus (d) regions. Mitochondrial density measured as % of COXIV intensity per tissue area in cortical grey matter (e), subcortical white matter (f), hippocampus CA1-CA3 (g), and dentate gyrus (h). Saline-control (*n* = 5 − 6), creatine-control (*n* = 5 − 6), saline-UCO (*n* = 7 − 8), and creatine-UCO (*n* = 6 − 7). Statistical analysis, two-Way ANOVA, and Tukey's multiple comparison. Data are shown as mean ± SEM analyzed by two-Way ANOVA, and Tukey's multiple comparison. Significance denoted by ^∗^ refers to *p* ≤ 0.05.

Representative images of COX-IV and cytochrome *c* oxidase staining are presented in Figure 4. Mitochondrial density was not significantly altered by UCO or creatine treatment in the grey matter, the subcortical white matter, or the CA1-CA3 and dentate gyrus regions of the hippocampus: (Figures 5(e)–5(h)). The ratio of cytochrome *c* oxidase in mitochondria to cytosol was not altered by UCO or creatine treatment in the cortical grey matter or subcortical white matter (Figures 5(a) and 5(b)). In contrast, in the CA1-CA3 region of the hippocampus, there was an interaction effect (Figure 5(c); *P*_INT=_ 0.01) where creatine treatment alone lowered this ratio in creatine-control fetuses and increased it following UCO, so that ratios were similar for the creatine-UCO fetuses and the saline-control fetuses. In the dentate gyrus region of the hippocampus, there was no effect of UCO or creatine treatment on the cytochrome *c* ratio; however, there was a trend for a UCO×treatment interaction (Figure 5(d), *P*_INT=_ 0.06).

### 3.4. Cell Death

**Figure 6:**
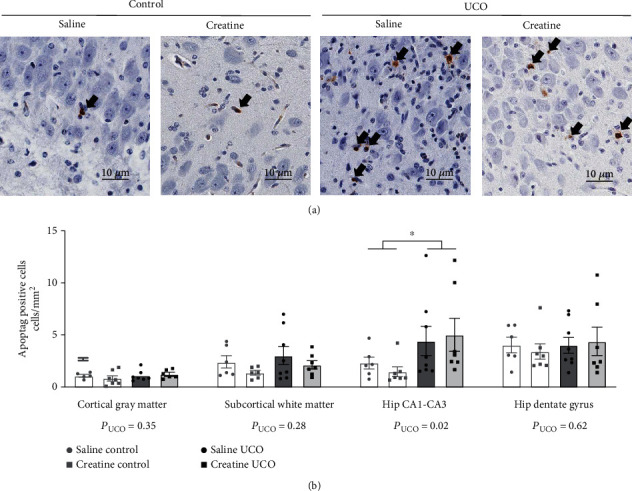
Apoptosis mediated cerebral cell death. Representative images of ApopTag staining the CA1-CA3 region of the hippocampus (a). Images are ×40 magnification. Scale bar represents 10 *μ*m. Black arrows point to positively stained cells. ApopTag-positive cells per area of mm^2^ in regions of the fetal brain (b). Saline-control (*n* = 6), creatine-control (*n* = 7), saline-UCO (*n* = 8), and creatine-UCO (*n* = 7). Statistical analysis, two-way ANOVA, and Tukey's multiple comparison. Data are mean ± SEM. Significance denoted by ^∗^ refers to *p* ≤ 0.05.

The ApopTag immunoassay allowed the quantification of cell death on fixed brain tissue sections (Figure 6(a)). At 72 hours following the 10-minute UCO, there were no differences in cell death counts in the cortical grey matter, subcortical white matter, or the dentate gyrus region of the hippocampus between treatment groups (Figure 6(b)). However, in the CA1-CA3 region of the hippocampus, UCO significantly increased cell death (Figure 6(b); *P*_UCO=_ 0.02), and the creatine treatment did not alter this outcome. No significant correlations were observed between cell death and total creatine content in the CA1-CA3 region or in the dentate gyrus region within the saline- or creatine-control fetuses (CA1-CA3 saline-control *r* = −0.37, *p* = 0.49 and creatine-control *r* = −0.07, *p* = 0.90; dentate gyrus saline-control *r* = −0.54, *p* = 0.29 and creatine-control *r* = 0.01, *p* = >0.99). However, in the dentate gyrus region, the creatine-UCO fetuses displayed a significant negative relationship between creatine tissue content and cell death counts (*r* = −0.82, *p* = 0.05), meaning that those fetuses with the highest creatine content after supplementation displayed the lowest cell death. In contrast, in the dentate gyrus region of saline-UCO fetuses, no significant correlation was observed between endogenous creatine content and cell death at 72 hours after the UCO (*r* = 0.25, *p* = 0.65). No significant correlation between creatine tissue content and cell death was observed in the grey or white matter regions.

### 3.5. Gene Analysis

Genes associated with cellular and mitochondrial markers of injury, mitogenesis, energy-sensing pathways, and creatine metabolism were assessed in the three fetal brain regions of interest at 72 hours post-UCO.

#### 3.5.1. mRNA Expression in the Cortical Grey Matter (Table 1)

Neither UCO nor creatine treatment significantly altered the mRNA expression of markers of cerebral injury including: apoptotic markers, vascular growth factor angiopoetin 2 (*ANGPT2*), and oxygen sensing *HIF1a*. In relation to mitophagy markers, the mRNA expression of the mitochondrial autophagy-related gene *Beclin-1* (BECN1) was significantly decreased following the UCO (*P*_UCO=_ 0.02), and this effect of UCO was reversed by creatine treatment (*P*_INT=_ 0.001). Multiple comparison revealed that the *BECN1* mRNA expression, a gene associated with autophagic-programmed cell death, was significantly downregulated in the saline-UCO fetuses compared to both the saline-control and creatine-UCO fetuses (*p* = 0.002 and *p* = 0.02, respectively). The mRNA expression of markers of mitochondrial structural integrity, *CRSL1* and *COX,* as well as mitochondrial density *(TRMT11:B2M*), was not altered by either UCO or creatine treatment. There was also no effect of UCO or creatine treatment on the mRNA expression of mitogenesis transcriptional regulators (*PCG1a*, and *TFAM*), profission genes (*DNML1*, *FIS1*, and *MFF*), or profusion genes (*OPA1*, *MFN1, MFN2*, and *MIEF1*). Lastly, neither UCO nor creatine treatment altered the mRNA expression of key energy homeostasis modulators (*ERRα*, *SIRT1*, and *SIRT3*) or genes associated with creatine metabolism (*SLC6A8* and *CKMT*).

#### 3.5.2. mRNA Expression in the Subcortical White Matter (Table 2)

When assessing markers of injury, a key finding in the subcortical white matter was the downregulation of vascular growth factor angiopoetin 2 (*ANGTP2*) mRNA expression by creatine treatment alone (*P*_TREAT=_ 0.02). There were no other significant changes in mRNA expression of cerebral markers of injury due to UCO or creatine treatment. When assessing mRNA expression of key mitochondrial markers of injury, creatine treatment significantly downregulated the *BECN1* mRNA expression in both the creatine-control and creatine-UCO fetuses (*P*_TREAT_ = 0.05). A similar effect was observed with the cytochrome c (*COX*) mRNA expression, where creatine treatment significantly downregulated the *COX* mRNA expression regardless of UCO (*P*_TREAT=_ 0.02). The mRNA expression of mitochondrial density ratio and mitogenesis regulators as well as fission and fusion genes was not altered by either UCO or creatine treatment in the subcortical white matter. Lastly, neither UCO nor creatine treatment significantly altered the mRNA expression of any of the assessed energy regulators or factors associated with creatine metabolism.

#### 3.5.3. mRNA Expression in the Hippocampus (Table 3)

The mRNA expression of the antiapoptotic gene *BCL2* was increased with creatine treatment (*P*_TREAT=_ 0.004) with no significant interaction effect between creatine treatment and UCO. All other markers of cerebral injury were not significantly altered by either UCO or creatine treatment. Creatine treatment downregulated the *BECN1* mRNA expression; however, this effect did not quite reach statistical significance (*P*_TREAT=_ 0.06), and *BECN1* was unaltered by UCO. Similarly, while the cardiolipin synthase 1 (CRLS1) mRNA expression was unaltered by UCO, it was significantly decreased with creatine treatment (*P*_TREAT=_ 0.03). There were no changes to the *COX* mRNA expression due to UCO or creatine treatment. In terms of transcriptional regulators of mitogenesis, the *TFAM* mRNA expression was significantly decreased by creatine treatment irrespective of UCO (*P*_TREAT=_ 0.01), whereas *PCG1a* was unaltered by both UCO and creatine treatment. The mRNA expression of the mitochondrial density ratio, fusion and fission genes, energy homeostasis modulators, and creatine metabolism factors was not altered by UCO or creatine treatment in the hippocampus. Lastly, the *ERRa* mRNA expression was upregulated by creatine treatment alone (*P*_TREAT=_ 0.01) but was unaltered by UCO.

## 4. Discussion

This study used a large animal model to characterize hypoxia-induced mitochondrial injury at a region-specific level in the fetal brain and to determine if creatine treatment could prevent or attenuate mitochondrial dysfunction. The main findings of this study were the presence of region-specific changes in mitochondrial function in the fetal brain following the mild hypoxic injury generated by UCO; specifically, 10 minutes of hypoxia resulted in the relative sparing of the cortical grey matter, while the periventricular white matter was more affected, and significant injury was detected in the hippocampus. In addition to the changes in respirometry parameters observed in the periventricular white matter and hippocampus, there was also clear evidence of increased cell death in the hippocampus CA1-3 region 72 hours after the hypoxic insult. These changes in the hippocampus and white matter region occurred in the absence of other mitochondrial and tissue injury, including cytochrome *c* release, altered mitochondrial density, expression of the ETC complexes, and mRNA expression of key markers of cellular injury. Therefore, the finding that mitochondrial respiration is impaired in the absence of extreme cellular injury suggests that mitochondrial respiration is particularly susceptible to in utero hypoxia in selected regions of the near-term fetal brain.This could increase vulnerability of these regions to subsequent hypoxic damage at birth or during neonatal resuscitation. While creatine treatment did not result in significant improvements in mitochondrial function, there were some modulatory effects of creatine across all three regions that can be seen as beneficial. Specifically, the negative relationship between cell death and creatine content in the hippocampus of supplemented fetuses suggests that creatine may support metabolism in this vulnerable region of the brain.

### 4.1. The Region-Specific Effects of UCO in the Near-Term Fetal Brain

In the current study, evidence of hypoxia-induced mitochondrial compromise and cell death was the greatest in the hippocampus. At 72 hours after UCO, all parameters of mitochondrial respiration were decreased, and in the CA1-CA3 region, apoptotic cell death was significantly increased. These findings are consistent with the known susceptibility of the hippocampus to hypoxic insult, which has also been observed in the human newborn brain after perinatal hypoxia at term [49, 50]. Another study using 10-minute cord occlusion in the near-term fetal sheep also reported significant injury in the hippocampus at 72 hours post-UCO [51]. Importantly, hypoxia-induced injury has been linked to mitochondrial dysfunction using electron microscopy, where transient mitochondrial swelling and abnormal crista structure were reported in the adult gerbil hippocampus immediately after 10 minutes of cerebral ischemia [35]. An adult rat study also reported decreased hippocampal mitochondrial respiration 48 hours after 30 minutes of forebrain ischemia [19].

A high constitutive metabolic demand has been identified as a characteristic that increases the susceptibility to hypoxic injury [10]. State I mitochondrial respiration reflects the basal aerobic metabolic activity in the presence of endogenous substrates and endogenous ADP [52, 53]. In the saline-control fetuses of the current study, the hippocampus displayed the highest state I respiration, which was 30% higher compared to the periventricular white matter and 36% higher compared to the cortical grey matter. Given that the hippocampus displayed more injury, our findings support the notion that a higher basal metabolic demand predisposes hippocampal neurons to hypoxic injury.

Despite the known vulnerability of the white and grey matter regions to hypoxic injury at term, this in utero study of hypoxia resulted in only moderate mitochondrial dysfunction in the periventricular white matter, while no injury was reported in the cortical grey matter. Previous studies by our group have shown significant evidence of injury at 48 hours following a 10-minute UCO in the near-term fetal sheep which produced more severe hypoxia, hypercapnia, and acidosis, resulting in lipid peroxidation, neuronal necrosis, diffuse grey and white matter lesions, hippocampal cell death, and abnormal vasculature changes [54–57]. However, in the cohort of pregnant sheep used here, UCO for 10-minutes produced only a mild hypoxic environment compared to these previous studies [32], consistent with the histological and gene assessments presented in this study. Nevertheless, the damage observed in the hippocampus and periventricular white matter illustrates the different vulnerability of these regions in the fetal brain to hypoxic injury. Also, the relative sparing of the cortical grey matter in this instance may be attributable to the presence of a network of small blood vessels known as pial vessels and their leptomeningeal anastomoses that may have sustained cortical perfusion during the 10 minutes of UCO [58, 59], so that severe grey matter injury appears as a characteristic of more profound hypoxia, e.g., when there is complete lack of oxygen for over 15 minutes [60]. It should also be noted that the current study selected grey matter from the outer cerebral hemisphere due to ease of rapid sampling. It is possible that the deeper grey matter regions (i.e. basal ganglia and thalamus) may have suffered some cellular damage, as these regions are known to be more vulnerable to hypoxic injury [61–63].

### 4.2. Implications of Reduced Mitochondrial Respiration in the Term Fetal Brain

Throughout gestation, mitochondrial function is important for brain development [22] and mitochondrial density and respiration are an important part of the transition to neonatal life [64]. While mitochondrial respiratory activity is low early in gestation due to a greater reliance on anaerobic glycolysis [64–66], during late gestation an increase in metabolic demand is accompanied by an increase in mitochondrial density and ETC protein abundance [17] with mitochondrial respiration markedly increasing even further immediately after birth [67]. This temporal discrepancy between mitochondrial respiration and density observed in late gestation is thought to be an adaptation to restricted oxygen consumption within an in utero environment, where oxygen supply is limited [67]. Therefore, any compromise of mitochondrial respiration by additional hypoxia late in gestation could lead to impaired mitochondrial ATP production and thus a reduced capacity to respond effectively to increased metabolic demands of birth.

In the hippocampus, the profound decrease in mitochondrial respiration post-UCO included a decrease in state I respiration. This initial steady-state measurement is controlled by cellular energy turnover and the degree of coupling to the rephosphorylation of endogenous ADP [68]. Previous studies have suggested that a reduction in steady-state respiration may be an adaptation to withstand the effects of a *prevailing* hypoxic state, because decreasing steady-state respiration may maintain metabolic requirements, and reduce superoxide production and oxidative stress within this hypoxic environment [66]. Importantly, a recent study reported that decreased mitochondrial respiration precedes cell death and suggested that lower basal respiration could be an adaptation to preserve energy in response to hypoxia [52]. The observation of a prolonged reduction in steady-state respiration at 72 hours after the hypoxic insult (and reestablishment of oxygen delivery) is therefore particularly significant and suggests that fetal mitochondrial function may not be able to recover from even a brief hypoxic challenge.

In both the hippocampus and the periventricular white matter, state III and state IV respiration were also significantly reduced by the UCO. State III represents ADP-stimulated respiration and is limited by the activity of the phosphorylation system. It also reflects the activity of ATP synthase and therefore ATP production [69]. Again, the finding that these changes of mitochondrial respiratory capacity are present so long after the brief and relatively mild hypoxia suggests that the near-term fetal brain may continue to be vulnerable to further injury, and if persisting, even have implications in the early postnatal period.

### 4.3. The Effects of Creatine Treatment on the Fetal Brain

While the dose and timing of creatine treatment used here did not rescue mitochondrial respiration deficits, creatine did have some effects in all three brain regions which can be construed to have been beneficial in the context of hypoxia. In the dentate gyrus region of the hippocampus, when creatine treated fetuses were subjected to UCO, there was a significant negative correlation between total creatine content and the incidence of cell death, i.e., the fetuses with the lowest cell death had the highest tissue creatine content: an outcome potentially related to the increased availability of creatine and a greater ability to maintain ATP during hypoxia and so reduce cell death [29, 70]. In comparison, no such relationship was observed for the saline-UCO fetuses. In the CA1-CA3 region of the hippocampus, creatine treatment also reduced cytosolic cytochrome *c* release, and the highest ratio of cytochrome *c* bound to the inner mitochondrial membrane was observed in the creatine-UCO fetuses, indicating greater mitochondrial structural integrity after the hypoxia caused by UCO. Also of note is that the prolonged treatment with creatine was not accompanied by cellular or mitochondrial changes except for an upregulation of mRNA expression of the antiapoptotic gene *Bcl2*. However, these effects of creatine following UCO on antiapoptotic gene expression did not ameliorate cell death in the CA1-CA3 region of the hippocampus following UCO. A plausible explanation for this is that although the extrinsic and intrinsic pathways of apoptosis are closely linked, they have been shown to be differentially regulated [71, 72]. Indeed, a study of brain cortical cell cultures from 14-day-old rat embryos found that creatine supplementation reduced intramitochondrial ROS generation, thus preserving mitochondrial membrane function and preventing the release of proapoptotic factors that activate caspases that then initiate the intrinsic apoptotic pathway [36]. Taken together, these findings suggest creatine has effects in regulating the intrinsic apoptotic pathway, but appeared to have no effects on the extrinsic pathway.

In terms of mitogenesis, creatine treatment alone upregulated the mRNA expression of *ERRα*, but downregulated *TFAM* mRNA in the hippocampus, a change unaffected subsequently by UCO. The importance of these changes is that *ERRα* [17] forms a complex with *PCG1-α*, the master regulator of mitogenesis, to drive mitochondrial biogenesis [73]. One downstream result of this is the upregulation of *TFAM*, which subsequently increases transcription of mtDNA-encoded proteins required for oxidative phosphorylation. An in vitro study on mouse myoblasts subjected to oxidative stress reported that creatine treatment significantly increased both *PCG1-α* and *TFAM* mRNA expression, suggesting that creatine stimulates mitogenesis [74]. While the overall results of this study do not confirm that creatine altered mitogenesis in the fetal sheep brain, the creatine treatment was associated with a trend for increased mitochondrial density and state IV respiration in the hippocampus. Collectively, these results suggest that supplementary creatine has a capacity to modulate mitogenesis; although, this relationship needs to be more thoroughly characterized by assessing the expression of key markers of mitogenesis at the protein level.

Creatine treatment also had effects on the *BECN1* mRNA expression following UCO in the cortical grey matter, in that it prevented the hypoxia-induced decrease in *BECN1* observed in the saline-UCO fetuses. *BECN1* eliminates damaged mitochondria to enhance cell survival [75]. Since *BECN1* upregulation has protective effects against ischemia-reperfusion injury in the kidney [76], the restoration of *BECN1* levels post-UCO mediated by creatine could be beneficial for cell survival.

### 4.4. Strength and Limitations of the Study Design

A strength of this study is the analysis of multiple aspects of mitochondrial function (i.e., respiration, mitogenesis transcription factors, and preliminary morphology results), which are not often reported together or within the context of mitochondrial function in fetal life. Moreover, the use of a large animal model enabled the characterization of these changes at a region-specific level within the fetal brain. However, a limitation of the study is the access to a relatively small number of animals and unequal sex distribution within groups, and that observations were made only at 72 hours after the UCO. The 72-hour time point was chosen based on previous histological assessment of brain pathology in this model of in utero hypoxia [37, 77, 78]. However, this provides only a single time point for assessment, and earlier effects on mitochondria might reveal the immediate and subsequent effects of the UCO, which are still unknown.

## 5. Conclusion

This study set out to identify if mitochondrial dysfunction in the fetal brain was present at 72 hours following a brief hypoxic event in utero near to term and to determine if creatine treatment was neuroprotective in this context. For the three brain regions analyzed in detail, the global fetal hypoxia induced a varying degree of cerebral injury with the greatest injury observed in the hippocampus. A functional decrease in mitochondrial respiration was observed in the hippocampus, and to a lesser extent in the periventricular white matter, in the absence of significant injury at a cellular and gene expression level, thus highlighting the vulnerability of mitochondria to a mild hypoxic injury in utero. While creatine treatment did not show strong evidence of neuroprotection, it did modulate markers of injury at both a cellular and gene expression level.

## Figures and Tables

**Table 1:** Grey matter mRNA gene analysis. Data expressed relative to the geomean of housekeeping genes (*RPS16*, *RPL32*, and *OAZ1*) and shown relative to saline-control fetuses. Saline-control: *n* = 4 − 6, creatine-control: *n* = 7, saline-UCO: *n* = 6 − 7, and creatine-UCO: *n* = 5 − 6. All data are expressed as mean ± SEM, analyzed by two-way ANOVA and Tukey's multiple comparisons test. Bolded text indicates statistically significant result (*p* < 0.05).

Subcortical grey matter		Control		UCO		Statistics		
		Saline	Creatine	Saline	Creatine	*P*_INT_	*P*_UCO_	*P*_TREAT_
Cerebral injury markers	*CASP3*	1.00 ± 0.20	1.03 ± 0.40	0.56 ± 0.24	0.95 ± 0.20	0.61	0.46	0.55
	*BAK*	1.00 ± 0.16	0.82 ± 0.54	0.45 ± 1.01	0.91 ± 0.09	0.72	0.74	0.34
	*BAX*	1.00 ± 0.04	1.04 ± 0.02	1.05 ± 0.07	1.03 ± 0.04	0.54	0.72	0.80
	*BCL2*	1.00 ± 0.17	0.73 ± 0.19	0.87 ± 0.42	1.30 ± 0.39	0.30	0.50	0.82
	*ANGTP2*	1.00 ± 0.08	0.94 ± 0.11	0.74 ± 0.17	0.63 ± 0.10	0.84	0.06	0.56
	*HIF1α*	1.00 ± 0.05	0.84 ± 0.06	0.93 ± 0.25	1.05 ± 0.15	0.41	0.67	0.94
	*BAX:BCL2*	1.25 ± 0.49	1.99 ± 0.62	2.13 ± 0.64	1.27 ± 058	0.22	0.92	0.89

Mito genes	*BECN1*	1.00 ± 0.04	0.81 ± 0.05	0.63 ± 0.05	0.89 ± 0.04	**<0.01**	**0.02**	0.51
	*CRLS1*	1.00 ± 0.13	1.04 ± 0.07	0.89 ± 0.06	0.88 ± 0.09	0.76	0.81	0.15
	*COX*	1.00 ± 0.05	0.90 ± 0.06	0.82 ± 0.07	0.98 ± 0.06	0.08	0.49	0.59

Mito density	*TRMT11:B2M*	1.05 ± 0.09	0.80 ± 0.11	0.98 ± 0.15	0.94 ± 0.18	0.53	0.74	0.41

Mitogenesis	*PCG1α*	1.00 ± 0.16	1.01 ± 0.11	0.67 ± 0.13	0.86 ± 0.07	0.50	0.08	0.45
	*TFAM*	1.00 ± 0.12	0.59 ± 0.09	0.81 ± 0.14	0.87 ± 0.12	0.09	0.70	0.20

Fission	*DNML1*	1.00 ± 0.03	0.84 ± 0.11	0.74 ± 0.08	0.91 ± 0.07	0.11	0.37	0.95
	*FIS1*	1.00 ± 0.15	1.37 ± 0.31	1.85 ± 0.50	1.28 ± 0.18	0.20	0.30	0.78
	*MFF*	1.00 ± 0.04	0.88 ± 0.08	1.23 ± 0.51	0.93 ± 0.05	0.78	0.67	0.54

Fusion	*OPA1*	1.00 ± 0.11	0.84 ± 0.11	0.67 ± 0.08	0.81 ± 0.06	0.17	0.10	0.96
	*MFN1*	1.00 ± 0.16	1.07 ± 0.08	0.77 ± 0.12	0.96 ± 0.141	0.66	0.21	0.33
	*MFN2*	1.00 ± 0.06	0.95 ± 0.12	0.61 ± 0.10	0.83 ± 0.15	0.31	0.06	0.49
	*MIEF1*	1.00 ± 0.06	0.88 ± 0.10	0.69 ± 0.06	0.78 ± 0.13	0.33	0.06	0.90

Energy metabolism	*SIRT1*	1.00 ± 0.07	0.91 ± 0.07	0.79 ± 0.06	1.00 ± 0.07	0.06	0.43	0.39
	*SIRT3*	1.00 ± 0.05	1.11 ± 0.10	0.93 ± 0.14	1.04 ± 0.04	0.96	0.50	0.30
	*ERRα*	1.00 ± 0.26	0.95 ± 0.14	1.65 ± 0.42	1.89 ± 0.72	0.72	0.06	0.80
	*PRKAA1*	1.00 ± 0.06	0.95 ± 0.12	0.82 ± 0.09	1.01 ± 0.13	0.33	0.62	0.56
	*PRKAA2*	1.00 ± 0.06	0.95 ± 0.12	0.67 ± 0.05	0.98 ± 0.10	0.12	0.17	0.20

Creatine metabolism	*SLC6A8*	1.00 ± 0.68	0.47 ± 0.25	0.31 ± 0.22	1.25 ± 1.05	0.22	0.93	0.71
	*CKMT*	1.00 ± 0.16	1.62 ± 0.25	1.02 ± 0.19	1.19 ± 0.21	0.36	0.40	0.11

**Table 2:** White matter mRNA gene analysis. Data expressed relative to the geomean of housekeeping genes (*RPS16*, *RPL32*, and *OAZ1*) and shown relative to saline-control fetuses. Saline-control: *n* = 5 − 6, creatine-control: *n* = 6 − 7, saline-UCO: *n* = 8, and creatine-UCO: *n* = 3 − 5. All data are expressed as mean ± SEM, analyzed by two-way ANOVA and Tukey's multiple comparisons test. Bolded text indicates statistically significant result (*p* ≤ 0.05).

Subcortical white matter		Control		UCO		Statistics		
		Saline	Creatine	Saline	Creatine	*P*_INT_	*P*_UCO_	*P*_TREAT_
Cerebral injury markers	*CASP3*	1.00 ± 0.21	3.62 ± 2.86	1.66 ± 0.81	2.74 ± 2.07	0.68	0.95	0.33
	*BAK*	1.00 ± 0.24	0.70 ± 0.18	1.02 ± 0.18	1.08 ± 0.45	0.49	0.42	0.64
	*BAX*	1.00 ± 0.08	0.80 ± 0.10	0.98 ± 0.05	0.90 ± 0.11	0.56	0.64	0.14
	*BCL2*	1.00 ± 0.38	0.60 ± 0.21	0.80 ± 0.15	1.15 ± 0.56	0.21	0.55	0.93
	*ANGTP2*	1.00 ± 0.16	0.53 ± 0.14	0.77 ± 0.10	0.50 ± 0.06	0.49	0.39	**0.02**
	*HIF1α*	1.00 ± 0.16	0.73 ± 0.10	1.00 ± 0.12	0.93 ± 0.12	0.52	0.50	0.27
	*BAX:BCL*	1.79 ± 0.56	1.86 ± 0.55	1.89 ± 0.51	1.75 ± 0.83	0.87	0.99	0.95

Mito genes	*BECN1*	1.00 ± 0.10	0.73 ± 0.09	1.04 ± 0.10	0.84 ± 0.06	0.75	0.49	**0.05**
	*CRLS1*	1.00 ± 0.09	0.98 ± 0.12	0.94 ± 0.06	1.01 ± 0.20	0.73	0.88	0.81
	*COX*	1.00 ± 0.09	0.87 ± 0.05	1.08 ± 0.05	0.85 ± 0.09	0.47	0.69	**0.02**

Mito density	*TRMT11:B2M*	1.79 ± 0.56	1.86 ± 0.55	1.89 ± 0.51	1.75 ± 0.83	0.87	0.99	0.95

Mitogenesis	*PCG1α*	1.00 ± 0.20	0.44 ± 0.08	1.02 ± 0.25	0.77 ± 0.15	0.50	0.43	0.08
	*TFAM*	1.00 ± 0.21	1.01 ± 0.19	0.96 ± 0.15	0.82 ± 0.15	0.72	0.59	0.77

Fission	*DNML1*	1.00 ± 0.16	0.56 ± 0.08	1.07 ± 0.13	0.90 ± 0.05	0.39	0.18	0.06
	*FIS1*	1.00 ± 0.87	0.57 ± 0.34	0.44 ± 0.15	2.14 ± 1.05	0.09	0.40	0.30
	*MFF*	1.00 ± 0.08	0.86 ± 0.06	1.05 ± 0.07	1.02 ± 0.01	0.51	0.21	0.33

Fusion	*OPA1*	1.00 ± 0.12	0.64 ± 0.11	1.01 ± 0.12	0.90 ± 0.13	0.39	0.35	0.13
	*MFN1*	1.00 ± 0.10	0.87 ± 0.13	0.96 ± 0.08	1.27 ± 0.12	0.09	0.14	0.47
	*MFN2*	1.00 ± 0.11	0.66 ± 0.10	0.93 ± 0.15	0.91 ± 0.24	0.35	0.58	0.31
	*MIEF1*	1.00 ± 0.12	0.76 ± 0.13	1.09 ± 0.18	0.81 ± 0.16	0.90	0.72	0.19

Energy metabolism	*SIRT1*	1.00 ± 0.13	0.83 ± 0.10	0.99 ± 0.10	1.04 ± 0.04	0.38	0.42	0.63
	*SIRT3*	1.00 ± 0.04	0.93 ± 0.08	1.01 ± 0.12	1.11 ± 0.18	0.51	0.88	0.44
	*ERRα*	1.00 ± 0.19	0.75 ± 0.32	1.62 ± 0.37	0.73 ± 0.24	0.37	0.41	0.13
	*PRKAA1*	1.00 ± 0.12	0.73 ± 0.09	1.08 ± 0.20	0.89 ± 0.10	0.84	0.48	0.20
	*PRKAA2*	1.00 ± 0.10	0.54 ± 0.11	0.88 ± 0.18	0.89 ± 0.10	0.18	0.49	0.19

Creatine metabolism	*SLC6A8*	1.00 ± 0.15	0.80 ± 0.14	1.05 ± 0.18	0.92 ± 0.27	0.87	0.68	0.46
	*CKMT*	1.00 ± 0.14	0.57 ± 0.13	1.34 ± 0.30	0.60 ± 0.18	0.60	0.52	0.06

**Table 3:** Hippocampus mRNA gene analysis. Data expressed relative to the geomean of housekeeping genes (*RPS16*, *RPL32*, and *OAZ1*) and shown relative to saline-control fetuses. Saline-control: *n* = 5 − 6, creatine-control: *n* = 6 − 7, saline-UCO: *n* = 7, and creatine-UCO: *n* = 5. All data are expressed as mean ± SEM, analyzed by two-way ANOVA and Tukey's multiple comparisons test. Bolded text indicates statistically significant result (*p* ≤ 0.05).

Hippocampus		Control		UCO		Statistics		
		Saline	Creatine	Saline	Creatine	*P*_INT_	*P*_UCO_	*P*_TREA__T_
Cerebral injury markers	*CASP3*	1.00 ± 0.49	2.22 ± 1.83	0.26 ± 0.08	0.55 ± 0.33	0.64	0.24	0.45
	*BAK*	1.00 ± 0.15	1.22 ± 0.10	0.79 ± 0.10	0.85 ± 0.05	0.91	0.07	0.07
	*BAX*	1.00 ± 0.08	0.98 ± 0.06	1.02 ± 0.03	0.93 ± 0.05	0.49	0.81	0.37
	*BCL2*	1.00 ± 0.25	1.41 ± 0.34	0.72 ± 0.17	2.28 ± 0.43	0.07	0.34	**<0.01**
	*ANGTP2*	1.00 ± 0.08	0.87 ± 0.17	0.75 ± 0.08	0.72 ± 0.10	0.67	0.12	0.51
	*HIF1α*	1.00 ± 0.12	1.07 ± 0.26	0.97 ± 0.08	1.17 ± 0.13	0.77	0.82	0.42
	*BAX:BCL2*	1.68 ± 0.76	1.29 ± 0.36	1.42 ± 0.34	0.51 ± 0.11	0.55	0.26	0.15

Mito genes	*BECN1*	1.00 ± 0.05	0.82 ± 0.08	0.95 ± 0.03	0.82 ± 0.12	0.78	0.74	0.06
	*CRLS1*	1.00 ± 0.09	0.74 ± 0.04	0.85 ± 0.07	0.77 ± 0.08	0.25	0.44	**0.03**
	*COX*	1.00 ± 0.05	1.00 ± 0.03	0.96 ± 0.06	0.89 ± 0.07	0.54	0.22	0.58

Mito density	*TRMT11:B2M*	1.06 ± 0.13	1.35 ± 0.52	1.10 ± 0.10	0.96 ± 0.17	0.49	0.56	0.80

Mitogenesis	*PCG1α*	1.00 ± 0.14	0.99 ± 0.15	1.09 ± 1.32	1.16 ± 0.16	0.79	0.38	0.81
	*TFAM*	1.00 ± 0.11	0.60 ± 0.11	0.83 ± 0.12	0.60 ± 0.11	0.50	0.50	**0.01**

Fission	*DNML1*	1.00 ± 0.06	0.96 ± 0.12	1.04 ± 0.05	1.09 ± 0.05	0.60	0.30	0.94
	*FIS1*	1.00 ± 0.22	1.96 ± 0.34	1.25 ± 0.21	1.44 ± 0.34	0.19	0.64	0.06
	*MFF*	1.00 ± 0.04	0.94 ± 0.06	0.99 ± 0.03	0.92 ± 0.09	0.88	0.88	0.28

Fusion	*OPA1*	1.00 ± 0.07	0.79 ± 0.09	0.98 ± 0.05	0.95 ± 0.12	0.31	0.40	0.19
	*MFN1*	1.00 ± 0.08	0.82 ± 0.08	0.88 ± 0.07	0.96 ± 0.12	0.18	0.87	0.60
	*MFN2*	1.00 ± 1.10	0.85 ± 0.15	1.02 ± 0.08	0.96 ± 0.20	0.77	0.65	0.48
	*MIEF1*	1.00 ± 0.03	0.89 ± 0.08	1.03 ± 0.09	0.94 ± 0.15	0.90	0.64	0.34

Energy metabolism	*SIRT1*	1.00 ± 0.06	0.86 ± 0.06	1.00 ± 0.07	0.88 ± 0.04	0.87	0.86	0.06
	*SIRT3*	1.00 ± 0.08	0.91 ± 0.10	1.06 ± 0.05	0.98 ± 0.10	0.99	0.44	0.35
	*ERRα*	1.00 ± 0.27	1.71 ± 0.34	1.33 ± 0.08	1.92 ± 0.22	0.80	0.27	**0.01**
	*PRKAA1*	1.00 ± 0.08	0.75 ± 0.09	0.96 ± 0.08	0.92 ± 0.13	0.32	0.50	0.18
	*PRKAA2*	1.00 ± 0.13	0.84 ± 0.12	0.99 ± 0.07	0.90 ± 0.16	0.80	0.85	0.33

Creatine metabolism	*SLC6A8*	1.00 ± 0.06	0.70 ± 0.10	1.02 ± 0.12	0.93 ± 0.18	0.40	0.14	0.32
	*CKMT*	1.00 ± 0.26	1.87 ± 0.97	0.91 ± 0.07	1.56 ± 0.45	0.85	0.74	0.20

## Data Availability

The raw data for this study could potentially be made available upon request to the corresponding author.

## Supplementary Materials

Supplementary Materials Table S1 provides preliminary data confirming the dosing regimen used in this study leads to significant fetal tissue creatine loading. Table S2 details the TaqMan® FAM™ labeled probes (including name and catalogue #) of genes selected for the Fluidigm assay in fetal brain samples. Figure S1 is a representative western blot of ETC complexes in fetal hippocampus. Figure S2 is a representative western blot of ETC complexes in fetal white matter.
