# Supplementary material for: The Effects of In Utero Fetal Hypoxia and Creatine Treatment on Mitochondrial Function in the Late Gestation Fetal Sheep Brain
Source: Oxid Med Cell Longev. 2022 Jan 29;2022:3255296. doi: 10.1155/2022/3255296 (PMC8817846; doi:10.1155/2022/3255296)
Supplement: Supplementary Materials — Table S1 provides preliminary data confirming the dosing regimen used in this study leads to significant fetal tissue creatine loading. Table S2 details the TaqMan® FAM™ labeled probes (including name and catalogue #) of genes selected for the Fluidigm assay in fetal brain samples. Figure S1 is a representative western blot of ETC complexes in fetal hippocampus. Figure S2 is a representative western blot of ETC complexes in fetal white matter. [file 3255296.f1.docx]

**Table S1. Total creatine content at 133 days gestation following 10 days of creatine infusion.** Preliminary studies conducted by Walker et al in 2017 confirmed that 10 days of direct fetal creatine infusion at 6 mg.kg^-1^.h^-1^ (diluted in saline and delivered at 1.5 mL/h) led to significant creatine loading in the fetal brain and other organs.

|  |  | **Saline**  **Infused (n=8)** | **Creatine Infused (n=8)** | **Statistics** |
| --- | --- | --- | --- | --- |
| **Tissue Creatine Content (mmol.kg^-1^DM)** | Brain | 45.1 ± 3.2 | 48.4 ± 2.3 | p<0.05 |
|  | Heart | 54.6 ± 4.5 | 60.6 ± 7.6 | pNS |
|  | Kidney | 13.7 ± 2.4 | 19.8 ± 4.4 | p<0.01 |
|  | Liver | 17.6 ± 1.5 | 20.2 ± 2.3 | p=0.01 |
|  | Placental Cotyledon | 6.7 ± 0.5 | 8.9 ± 0.8 | p<0.001 |
|  | Gastrocnemius Muscle | 84.1 ± 2.9 | 91.4 ± 3.6 | p<0.001 |

Data are presented as mean ± SD. Statistics paired students t-test. P < 0.05 was considered as statistically significant.

**Table S2.** Genes selected for the Fluidigm assay in fetal brain samples, including details on the TaqMan^®^ FAM™ labelled probes (name and catalogue #) used for the analyses.

## **Figure S1.** **Representative western blot of ETC complexes in fetal hippocampus**, in two separate gels. Positive control sample is labelled as P. Each complex is imaged at optimal intensity.


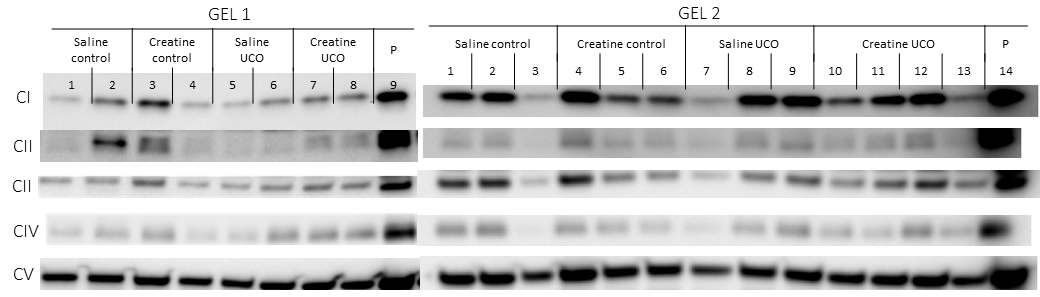


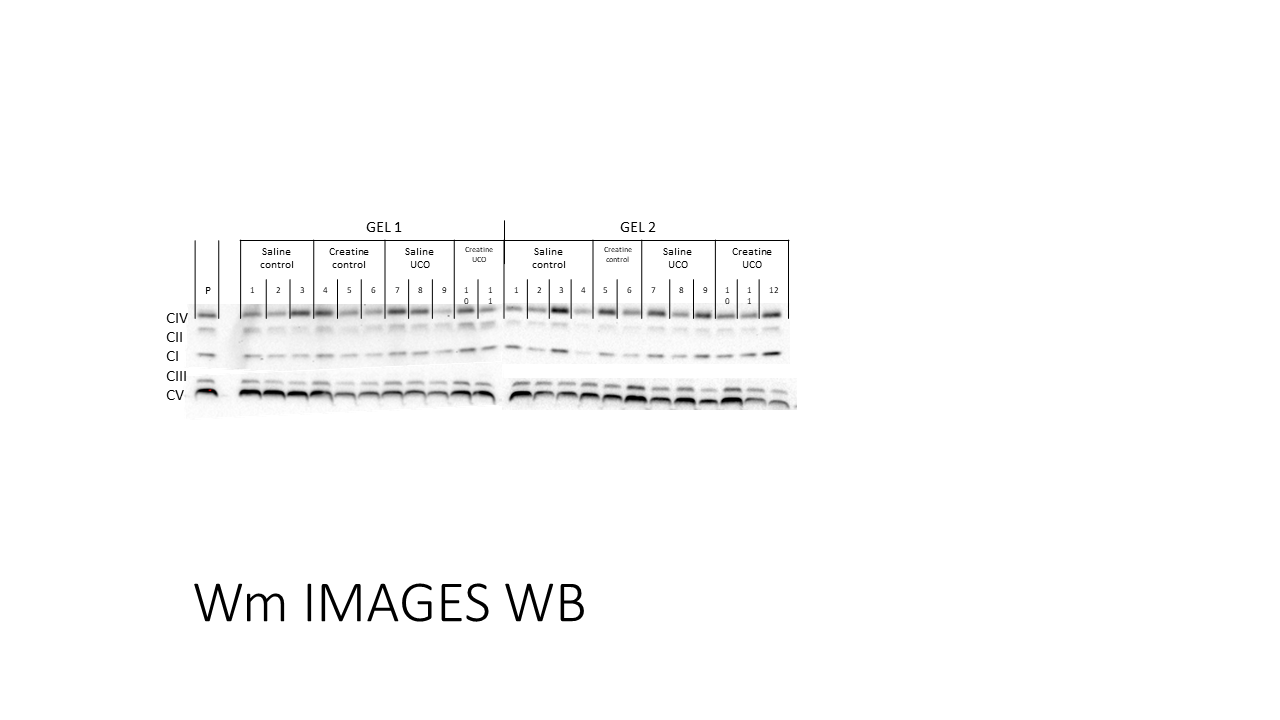
**Figure S2. Representative western blot of ETC complexes in fetal white matter**, in two separate gels. Positive control sample is labelled as P. Each complex is imaged at optimal intensity.
